# The Biogeographic Pattern of Microbial Functional Genes along an Altitudinal Gradient of the Tibetan Pasture

**DOI:** 10.3389/fmicb.2017.00976

**Published:** 2017-06-13

**Authors:** Qi Qi, Mengxin Zhao, Shiping Wang, Xingyu Ma, Yuxuan Wang, Ying Gao, Qiaoyan Lin, Xiangzhen Li, Baohua Gu, Guoxue Li, Jizhong Zhou, Yunfeng Yang

**Affiliations:** ^1^State Key Joint Laboratory of Environment Simulation and Pollution Control, School of Environment, Tsinghua UniversityBeijing, China; ^2^Key Laboratory of Alpine Ecology and Biodiversity, Institute of Tibetan Plateau Research, Chinese Academy of SciencesBeijing, China; ^3^CAS Center for Excellence in Tibetan Plateau Earth ScienceBeijing, China; ^4^Department of Earth System Science, Tsinghua UniversityBeijing, China; ^5^Department of Earth and Atmospheric Sciences, University of Houston, HoustonTX, United States; ^6^Key Laboratory of Adaptation and Evolution of Plateau Biota, Northwest Institute of Plateau Biology, Chinese Academy of SciencesXining, China; ^7^Key Laboratory of Environmental and Applied Microbiology, Environmental Microbiology Key Laboratory of Sichuan Province, Chengdu Institute of Biology, Chinese Academy of SciencesChengdu, China; ^8^Environmental Sciences Division, Oak Ridge National Laboratory, Oak RidgeTN, United States; ^9^College of Resources and Environmental Science, China Agricultural UniversityBeijing, China; ^10^Institute for Environmental Genomics and Department of Microbiology and Plant Biology, University of Oklahoma, NormanOK, United States; ^11^Earth Sciences Division, Lawrence Berkeley National Laboratory, BerkeleyCA, United States; ^12^Collaborative Innovation Center for Regional Environmental Quality, School of Environment, Tsinghua UniversityBeijing, China

**Keywords:** microbial biogeography, soil microbial community, microbial functional potential, alpine grassland, altitudinal gradient

## Abstract

As the highest place of the world, the Tibetan plateau is a fragile ecosystem. Given the importance of microbial communities in driving soil nutrient cycling, it is of interest to document the microbial biogeographic pattern here. We adopted a microarray-based tool named GeoChip 4.0 to investigate grassland microbial functional genes along an elevation gradient from 3200 to 3800 m above sea level open to free grazing by local herdsmen and wild animals. Interestingly, microbial functional diversities increase with elevation, so does the relative abundances of genes associated with carbon degradation, nitrogen cycling, methane production, cold shock and oxygen limitation. The range of Shannon diversities (10.27–10.58) showed considerably smaller variation than what was previously observed at ungrazed sites nearby (9.95–10.65), suggesting the important role of livestock grazing on microbial diversities. Closer examination showed that the dissimilarity of microbial community at our study sites increased with elevations, revealing an elevation-decay relationship of microbial functional genes. Both microbial functional diversity and the number of unique genes increased with elevations. Furthermore, we detected a tight linkage of greenhouse gas (CO_2_) and relative abundances of carbon cycling genes. Our biogeographic study provides insights on microbial functional diversity and soil biogeochemical cycling in Tibetan pastures.

## Introduction

It is estimated that two–thirds of the Tibetan plateau, the highest and largest plateau on earth characterized by cold climate, low oxygen level, strong UV irradiation and poor primary productivity, are comprised of alpine grasslands (Qiu, 2008). In the past decade, Tibet has witnessed more than three-fold increases in average Gross Domestic Product per capita and a 10% increase in human population, resulting in pronounced influence on natural environments (National Bureau of Statistics of China, 2015-2006). In addition, global warming has led to the substantial glacier and permafrost thaw, which impose risks of drought and flooding (Qiu, 2008). Since soil is considered to be the most complex ecosystem on earth and plays a vital role in mediating biogeochemical cycling (Falkowski et al., 2008; Bardgett and van der Putten, 2014), it is of great interest to examine the biogeography of soil microbial communities in such a fragile ecosystem (Wu et al., 2016a).

To date, biogeographic variations of microbial communities have predominantly been studied at the taxonomic level (Bryant et al., 2008; Singh et al., 2012). Phylogenetic diversities along elevation gradients showed a dip pattern (Singh et al., 2014) or a bulge pattern (Singh et al., 2012, 2016) at mid elevations, a monotonic decrease pattern (Bryant et al., 2008), or no pattern (Lauber et al., 2009; Fierer et al., 2011). The major controlling factors in driving the phylogenetic diversity can be attributed to temperature (Nottingham et al., 2016), soil pH (Zhang et al., 2015), C/N (Siles and Margesin, 2016), carbon content, climate variability (Delgado-Baquerizo et al., 2016) and plant diversity (Zhang et al., 2015). Nonetheless, there is a renewed interest in understanding the biogeography of microbial functional traits, which sheds light on microbial community functions (Green et al., 2008). GeoChip, a microarray-based tool, is excellent in detecting microbial functional genes. It contains DNA probes targeting a variety of functional genes, including those associated with elemental cycling, stress response, metal resistance and complex carbon degradation (He et al., 2007). It has been successfully used to profile functional genes of microbial communities in different habitats (Van Nostrand et al., 2009; Chu et al., 2014; Ding et al., 2015), which provides important insights into microbe-mediated processes. It was shown that measurements of relative abundances of functional genes were predictive of *in situ* CO_2_ and N_2_O emissions (Morales et al., 2010) and nitrification potential (Zhao et al., 2014) to certain extent.

Recently, we have used GeoChip to examine functional genes of soil microbial communities in an enclosed Tibetan alpine grassland to prevent anthropogenic disturbance, which revealed strikingly tight linkages between microbial functional gene structure and several environmental variables (Yang et al., 2014). Livestock grazing is the most prevalent economic activity in Tibet (Zhou et al., 2006), resulting in significant ecological effects. For example, it reduces vegetation biomass, switches dominant plant species to short and prostrate forb species (Lkhagva et al., 2013), expands the range of soil temperature fluctuation, increases soil mean temperature, enhances forage quality (Odriozola et al., 2014), and decreases microbial biomass carbon and nitrogen (Fu et al., 2012). Furthermore, owing to strong ruminant CH_4_ emission, total greenhouse gas budget of high grazing densities (-1034 g CO_2eq_ m^-2^ yr^-1^) was nearly four times to that of low grazing densities (-260 g CO_2eq_ m^-2^ yr^-1^) (Skiba et al., 2013). Thus, it is important to examine microbial biogeography of functional genes in the grassland subjected to livestock grazing, which is more common in Tibet.

In this study, we collected soil samples along an altitudinal gradient of 3200, 3400, 3600, and 3800 m asl in a Tibetan grassland subjected to open free livestock grazing. We adopted GeoChip 4.0 to examine microbial functional genes, aiming to address the following hypotheses: (i) because vegetation removal, manure deposition, and trampling by grazing impose multi-dimensional disturbance to soil, microbial biogeographic pattern in pasture would substantially differ from what is observed in the enclosed grassland; (ii) the relative abundance of specific microbial functional genes can be used to explain CO_2_ and N_2_O fluxes. The relationship between gene abundance and process rates has been a topic of dispute. It was shown recently that the activity of C degradation enzymes (ß-D-cellulosidase, ß-Xylosidase, *α*-Glucosidase and N-acetyl-ß-Glucosaminidase) in grain-producing soils was significant correlated to the relative abundance of microbial functional gene abundance determined by GeoChip 4.0 (*R*^2^> 0.90, *P* < 0.001) (Trivedi et al., 2016), and *amoA* gene relative abundances had significant positive correlations with soil nitrification potentials (*R* > 0.48, *P* < 0.05) in agricultural soils (Liu et al., 2015). However, a meta-analysis showed that the linkages between gene abundance and processes are often missed in many studies, which can be ascribed to habitat type or differences among genes (Rocca et al., 2015). Therefore, it is interesting to examine whether there is such linkage in this study.

## Materials and Methods

### Site Description and Soil Sampling

This study was carried out at the Haibei Alpine Meadow Ecosystem Research Station located in the northeast of the Tibetan Plateau. The region has a typical plateau continental climate characterized by cold and long winter but warm and short summer, with annual mean air temperature of -1.7°C and annual mean precipitation of 570 mm (Zhao et al., 2006). The vegetation growing season is from May to September. The major soil type is *Mat Cryic Cambisol* based on Chinese Soil Taxonomic Classification System (Arthur et al., 2008).

Soil samples were collected at elevations of 3200, 3400, 3600, and 3800 m asl. Geographical distances between adjacent elevations were 1.3 km (3600–3800 m), 4.2 km (3400–3600 m), and 6.2 km (3200–3400 m). To reduce soil heterogeneity, five soil cores at the 0–10 cm and 10–20 cm depths were randomly taken from a location with a size of 1 × 1 m and thoroughly mixed to make a composite sample. Three such locations at each elevation, which were several to dozens of meters away from each other, were chosen to create biological triplicates. All soil samples were collected in August 2009 and immediately transported on ice to the laboratory. After being sieved with 2 mm mesh to remove visible vegetation roots, residues and stones, parts of samples were stored at -80°C for DNA extraction and 4°C for environmental variable measurements, respectively.

### DNA Extraction, Purification, and Quantitation

After mixing soil cores from 0 to 10 cm and 10 to 20 cm depths, we extracted soil DNA with FastDNA spin kits (MP Biomedical, Carlsbad, CA, United States) following manufacturer’s instructions and then purified the extracted DNA in a mixture of 2.0 volume of 100% ice-cold ethanol and 0.1 volume of 3 M NaOAc (pH 5.2). After overnight incubation at -20°C, DNA solutions were centrifuged at 13000 *g* for 30 min and then washed with 1 ml 70% ethanol. Then DNA solutions were air dried for 30 min and dissolved in nuclease-free water. We used NanoDrop ND-1000 spectrophotometer (NanoDrop Technologies Inc., Wilmington, DE, United States) to determine DNA quality and a PicoGreen method using a FLUOstar Optima (BMG Labtech, Jena, Germany) to determine DNA concentrations (Ahn et al., 1996).

### GeoChip 4.0 Experiments

DNA was labeled with Dye Cy-5 by a random priming method as previously described (Yang et al., 2017) and the labeled DNA was purified with QIA quick purification kits (Qiagen, Valencia, CA, United States). SpeedVac (ThermoSavant, Milford, MA, United States) was used to dry DNA at 45°C for 45 min. We hybridized DNA with GeoChip 4.0 on a MAUI hybridization station (BioMicro, Salt Lake City, UT, United States) at 42°C for 16 h, as described previously (Yue et al., 2015). The slides were scanned using a NimbleGen MS200 scanner (Roche, Madison, WI, United States) at 633 nm using 100% laser power and 75% photomultiplier tube gain. Signal intensities were quantified by scanned images.

### The Elevation-Decay Relationship (EDR)

Sorensen similarity was calculated for generating the EDR *z*-value. An equation of log(*S*_S_) = constant-2*z*log(*D*) was used to calculate power-law exponent *z*, in which *D* was the Euclidean distance between two elevations and *S*_S_ was the pairwise similarity in microbial compositions by using Sorensen’s index. Elevation differences for samples at the same elevation were zero, which were changed into 0.01 to enable logarithm transformation (Legendre and Legendre, 1998). Since our pairwise comparison data were not independent with each other, we determined whether the slope was significant different from zero by bootstrapping (999 times) (Efron and Tibshirani, 1994). To this end, the original dataset was randomly paired (with replacement) and the slope of EDR was calculated by the new dataset. Using one sample *t*-test, we determined whether the slopes of the regressions based on bootstrapping differed from the observed slope followed by BH correction for *P*-value (Zhou et al., 2008).

### Data Analysis

We processed raw data as follows: (i) removing spots with a signal to noise ratio of less than 2.0; (ii) removing spots detected only once in three replicates; (iii) normalizing intensity of each spot by dividing total signal intensity of a sample and multiplying by a constant; and (iv) taking natural logarithm (Wu et al., 2016b). Subsequently, the detrended correspondence analysis (DCA), a multivariate statistical method (Ayoub-Hannaa et al., 2013), was used to compare overall functional gene structures across different samples. The hierarchical clustering analysis was used to cluster different microbial functional gene structures among soil samples. The dissimilarity test of adonis, one of the permutational multivariate analysis using distance matrices (Ricotta and Burrascano, 2009), was used to test differences of functional gene structures among different elevations. Alpha diversities were presented by Shannon and Simpson indexes. The canonical correspondence analysis (CCA) was used to identify main environmental drivers in shaping microbial communities. Variance inflation factors (VIF) of less than 20 were used as a threshold to remove redundant variables before performing CCA. The variation partitioning analysis (VPA) was used to partition environmental variables selected by CCA into three groups. Simple Mantel tests were performed to determine the relationships between microbial communities and environmental variables. Correlations between relative abundances of functional genes and environmental variables were determined by Pearson correlation and then adjusted by false discovery rate (FDR) method. These analyses were carried out using functions in the vegan package (v. 2.3-1) in R-studio v. 3.2.2. Association network analysis was performed with a subset of GeoChip data since the whole dataset was beyond the computational capacity. A random matrix theory (RMT)-based algorithm was used to construct networks using the coefficients of Spearman correlations to define similarity matrix (Deng et al., 2012).

### Measurements of Soil and Vegetation Variables

We recorded *in situ* soil temperatures using type-K thermocouples (Campbell Scientific, Logan, UT, United States) with a CR1000 datalogger at the depth of 10 cm, which represents 0–20 cm. We measured *in situ* soil moisture at the depth of 10 cm using a time domain reflectometry (Model Diviner-2000, Sentek Pty Ltd., Stepney, SA, Australia). Soil pH data were missing for our sample. Therefore, we used soils nearby our plots collected in 2012 as substitutes since soil pH in Tibet was a generally stable variable, which had decreased only by 0.5 units since 1980 (Zhou et al., 2009). We measured soil pH with a combination glass electrode in soil/water solution of volume ratio 1:5. The concentrations of ammonia and nitrate were detected by a FIAstar 5000 Analyzer (Yang et al., 2013). To generate finer measurements of nitrogen, carbon and phosphorus, we examined total nitrogen (TN), total organic carbon (TOC) and total phosphorus at both depths of 0–10 cm and 10–20 cm. We determined TN by a Vario EL III Elemental Analyzer (Elementar, Hanau, Germany), TOC by a TOC-5000A analyzer (Shimadzu Corp., Kyoto, Japan) and total phosphorus by a UV-visible spectrophotometer (Agilent 8453, Agilent Co., Santa Clara, CA, United States). Using a Agilent 4890D gas chromatograph (Agilent Co., Santa Clara, CA, United States) equipped with Flame Ionization Detector (FID) and Electron Capture Detector (ECD) (Wang and Wang, 2003), we quantified greenhouse gasses of CO_2_, CH_4_ and N_2_O except the 3600 m site, where rat holes were too rampant to perform accurate measurements of greenhouse gasses (Yang et al., 2014). During gas sampling, static stainless steel chambers (40 cm × 40 cm × 40 cm) were manually closed for half an hour. Then gas samples were taken every 10 min by plastic syringes and sampled within 24 h (Hu et al., 2010). We determined vegetation variables of species number, aboveground vegetation biomass and vegetation diversity (Shannon index).

## Results

### Environmental Variables

Soil temperature (T) and pH decreased along the altitudinal gradient (**Table 1**), while soil moisture, nitrate (NO_3_^-^) and TN measured at the 10–20 cm depth (TN_10-20cm_) increased with elevations. TOC measured at the 10–20 cm depth (TOC_10-20cm_), TN measured at the 0–10 cm depth (TN_0-10 cm_) and vegetation diversity were higher at 3400 and 3600 m than those at 3200 and 3800 m. Notably, there were correlations (*P* < 0.050) among TOC, TN, and total phosphorous (TP) (Supplementary Table S1).

**Table 1:** Summary of environmental variables.

Environmental variable	3200 m samples	3400 m samples	3600 m samples	3800 m samples
*T* (°C)^a^	12.42	9.15	8.87	6.84
pH	7.88 ± 0.11ab	7.73 ± 0.10a	7.01 ± 0.16b	7.17 ± 0.13b
Moisture (%)	25.84 ± 1.57d	31.66 ± 1.94c	53.78 ± 4.05a	42.99 ± 3.32b
NO_3_^-^ (mg kg^-1^)	0.94 ± 0.16b	0.66 ± 0.21b	1.40 ± 0.52a	1.54 ± 0.19a
NH_4_^+^ (mg kg^-1^)	2.43 ± 0.13b	2.89 ± 0.25a	2.22 ± 0.20b	1.34 ± 0.29c
TOC_0-10 cm_ (%)	6.24 ± 0.06b	7.29 ± 0.03a	6.13 ± 0.05b	5.53 ± 0.06c
TOC_10-20 cm_ (%)	2.27 ± 0.11c	5.20 ± 0.05a	5.30 ± 0.04a	4.85 ± 0.08b
TN_0-10 cm_ (g kg^-1^)	4.61 ± 0.06c	5.72 ± 0.07a	5.07 ± 0.03b	4.63 ± 0.07c
TN_10-20 cm_ (g kg^-1^)	2.99 ± 0.03d	3.72 ± 0.03c	4.01 ± 0.04b	4.25 ± 0.06a
TP_0-10 cm_ (g kg^-1^)	0.53 ± 0.01d	0.90 ± 0.02a	0.66 ± 0.01b	0.60 ± 0.01c
TP_10-20 cm_ (g kg^-1^)	0.36 ± 0.01a	0.57 ± 0.02a	1.97 ± 2.62a	0.42 ± 0.02a
SIN (mg kg^-1^)	3.37 ± 0.07ab	3.56 ± 0.34a	3.62 ± 0.54a	2.88 ± 0.19b
Vegetation biomass (g)	432.28 ± 18.02b	471.07 ± 22.93a	238.99 ± 6.52c	229.12 ± 17.07c
Vegetation diversity^c^	1.91 ± 0.09d	2.58 ± 0.05a	2.36 ± 0.04b	2.06 ± 0.08c
Vegetation species number	22.00 ± 0.88b	26.26 ± 1.50a	22.93 ± 1.10b	19.46 ± 0.68c
CH_4_ flux (μg m^-2^h^-1^)	–27.79 ± 14.04ab	–37.38 ± 8.98b	–	–13.66 ± 8.97a
CO_2_ flux (μg m^-2^h^-1^)	879.39 ± 59.85a	771.84 ± 108.29a	–	272.68 ± 40.81b
N_2_O flux (μg m^-2^h^-1^)	15.67 ± 5.45a	5.95 ± 11.49a	–	4.59 ± 1.64a

*^a^Abbreviations: T, soil temperature; TOC, total organic carbon; TN, total nitrogen; TP, total phosphorus; SIN, soil inorganic nitrogen; 0–10 and 10–20 cm, soil measured at 0–10 and 10–20 cm depths, respectively. ^b^Significant differences among elevations are indicated by different alphabetic letters. ^c^Vegetation diversity was calculated by Shannon index.*

### The Biogeographic Pattern of Microbial Functional Genes

A total of 52864 genes were detected by GeoChip, ranging from 31528 to 43816 for each site (Supplementary Table S2). The results of the dissimilarity test of *adonis* showed that samples were grouped by elevations (Supplementary Table S3). Microbial functional diversities, represented as Shannon indices, ranged in 10.27–10.58 and increased (*P* < 0.050) with elevations (Supplementary Table S2). However, the evenness indices of microbial functional genes were similar across elevations (Supplementary Table S2).

### The Elevation-Decay Relationship (EDR)

**FIGURE 1:**
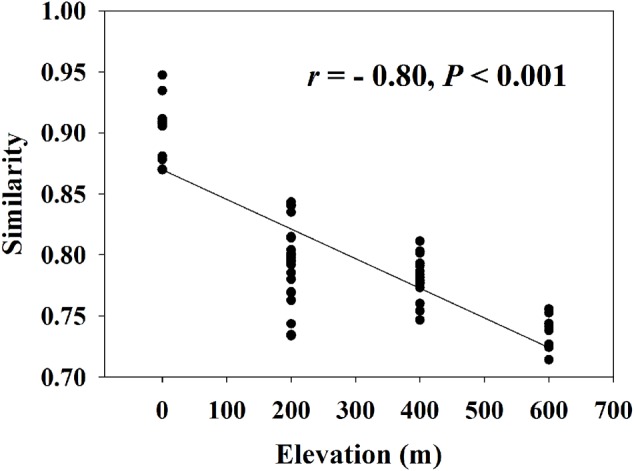
Elevation-decay relationship for whole microbial community (*z*-value = 0.0093, *t* = –209.6, *n* = 999, *P* < 0.001). Similarity represents Sorensen’s similarity index.

A significant EDR (*r* = -0.80, *P* < 0.001) was observed for the whole functional community (**Figure 1**) with a *z*-value of 0.0093 (Supplementary Table S4). We found that almost all *z*-values of carbon or nitrogen cycling processes were lower than 0.01, with the highest *z*-value (0.0127) of anammox and the lowest *z*-value (0.0055) of nitrogen fixation.

Ubiquitous genes, defined as genes detected in all of four elevations, accounted for 50.41% of all genes detected by GeoChip (Supplementary Figure S1). In contrast, unique genes, defined as genes detected only in one elevation, accounted for only 20.98% of all detected genes (Supplementary Figure S1). The number of unique genes increased with elevations, which was in accordance with the increasing ratio of the unique gene number to the detected gene number at each elevation (Supplementary Table S5). The percentage of shared genes between different elevations was the highest between 3600 and 3800 m asl (65.47%), which was the shortest in distance (Supplementary Table S6). In contrast, it was the lowest between 3200 and 3800 m asl (54.57%), which was the highest in distance. A negative correlation between distance and the number of shared genes (*r* = -0.71, *P* < 0.001) (Supplementary Figure S2) was detected.

### The Linkage of Overall Microbial Functional Gene Structure and Environmental Variables

We performed simple Mantel tests to examine major factors linking to overall microbial functional gene structure. The results showed that elevation was the most important factor in affecting functional genes structure (**Table 2**). To discriminate whether elevational influence is attributable to environmental variables, we performed CCA to determine the linkage between microbial functional gene structure and environmental variables (Supplementary Figure S3). Seven environmental variables (T, pH, NO_3_^-^, TP_10-20cm_, aboveground vegetation biomass, vegetation species number and vegetation diversity), were identified to have a significant influence on functional microbial structure, which was also verified by Mantel tests (*P* < 0.05) (**Table 2**). The VPA showed that these seven variables explained a total of 82.64% variations of functional gene structure (Supplementary Figure S4).

**Table 2:** Simple Mantel tests between functional genes and environmental variables.

Environmental variable	*R*	Environmental variable	*r*
Elevation (m)	0.80^∗∗∗b^	TP_0-10 cm_ (g kg^-1^)	0.10
*T*^a^ (°C)	0.65^∗∗∗^	TP_10-20 cm_ (g kg^-1^)	0.005
pH	0.51^∗∗∗^	SIN (mg kg^-1^)	0.35^∗∗^
Moisture (%)	0.52^∗∗^	Vegetation biomass (g)	0.52^∗∗^
NO_3_^-^ (mg kg^-1^)	0.29^∗^	Vegetation diversity	0.14
NH_4_^+^ (mg kg^-1^)	0.56^∗∗∗^	Vegetation species number	0.27^∗^
TOC_0-10 cm_ (%)	0.38^∗^	CH_4_ flux (μg m^-2^h^-1^)	0.19
TOC_10-20 cm_ (%)	0.28^∗^	CO_2_ flux (μg m^-2^h^-1^)	0.58^∗∗∗^
TN_0-10 cm_ (g kg^-1^)	0.11	N_2_O flux (μg m^-2^h^-1^)	0.06
TN_10-20 cm_ (g kg^-1^)	0.56^∗∗^		

*^a^Abbreviations: T, soil temperature; TOC, total organic carbon; TN, total nitrogen; TP, total phosphorus; SIN, soil inorganic nitrogen; 0–10 and 10–20 cm, environmental variables measured at 0–10 and 10–20 cm depths, respectively. ^b^Significance of differences: ^∗∗∗^*P* < 0.001, ^∗∗^*P* < 0.010, ^∗^*P* < 0.050.*

### Carbon Cycling Genes

Almost all of carbon cycling genes (Supplementary Figure S5) increased in relative abundance with elevations. However, there are certain exceptions. The relative abundances of *pulA* genes (encoding extracellular polyurethane/esterase) (Supplementary Figure S5A), and *rubisco* genes (encoding ribulose-1, 5-bisphosphate carboxylase/oxygenase) were lowest at the 3800 m site (Supplementary Figure S5B).

**FIGURE 2:**
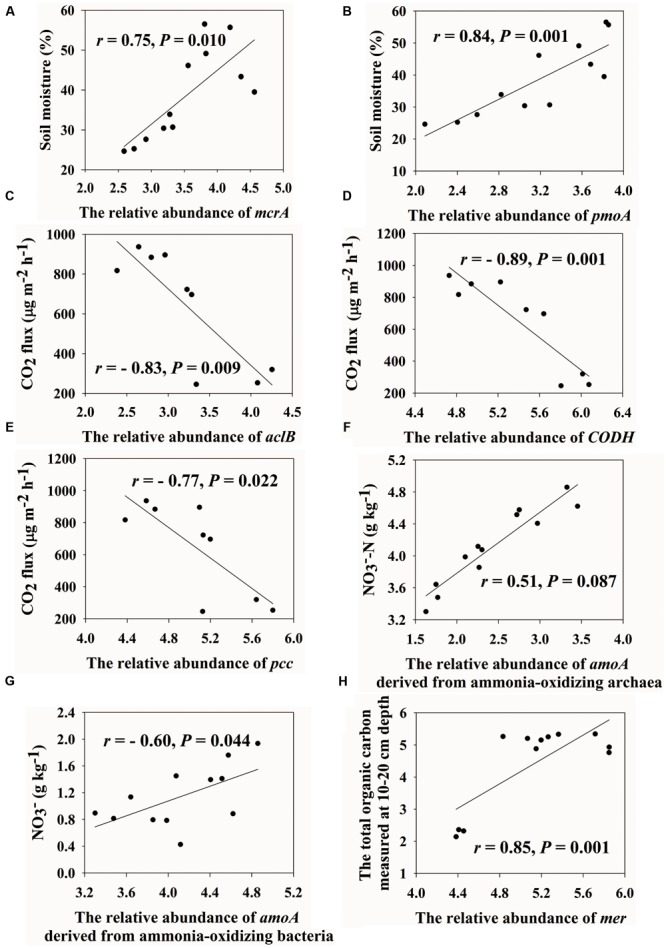
Correlations between soil moisture and the relative abundance of *mcrA* **(A)** and *pmoA* **(B)**, CO_2_ flux and the relative abundance of *aclB* **(C)**, *CODH* **(D)** and *pcc* **(E)**, nitrate and the relative abundance of *amoA* drived from ammonia-oxidizing archaea **(F)** and bacteria **(G)** and the total organic carbon measured at 10–20 cm depth and the relative abundance of mer **(H)**. Pearson correlation and TDIST tests were used to calculate *r* and *P* values, respectively.

We found that soil moisture has a strong positive correlation with *mcrA* genes (encoding methyl coenzyme M reductase subunit A, *r* = 0.75, *P* = 0.010) and *pmoA* genes (encoding particulate methane monooxygenase A, *r* = 0.84, *P* = 0.001) (**Figures 2A,B**). In addition, the important role of microbes on CO_2_ flux was verified by Mantel tests (*r* = 0.58, *P* < 0.001) (**Table 2**). In-depth investigation showed that CO_2_ flux negatively correlated with *aclB* genes (encoding ATP citrate lyase involved in reductive TCA cycle, *r* = -0.83, *P* = 0.009), *CODH* genes (encoding carbon monoxide dehydrogenase involved in biosynthesis of acetyl-CoA, *r* = -0.89, *P* = 0.001) and *pcc* genes (involved in hydroxypropionate pathways, *r* = -0.77, *P* = 0.022) (**Figures 2C–E**).

**FIGURE 3:**
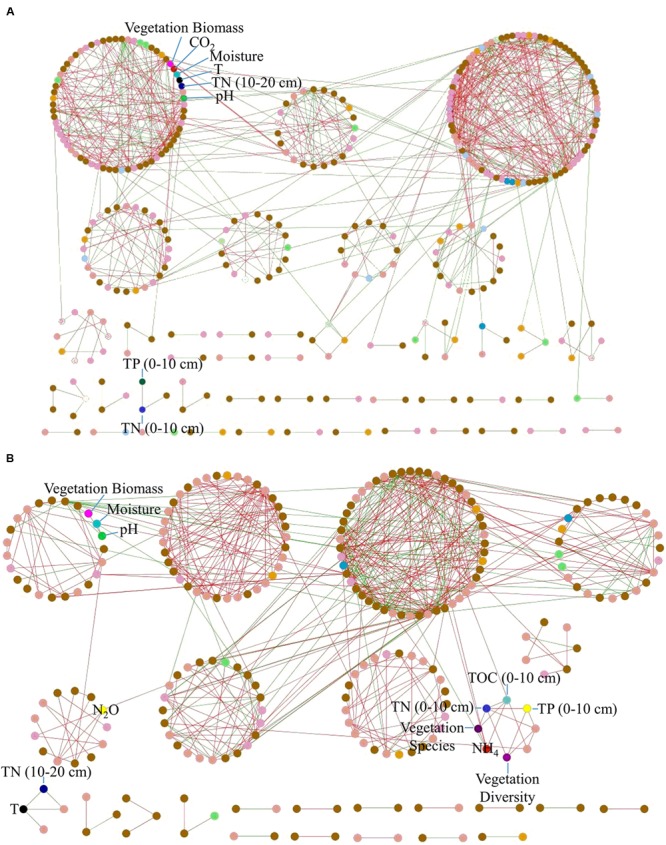
Association network analyses of **(A)** carbon fixation genes and **(B)** *amoA* genes.

To identify major carbon fixation bacteria responsible for CO_2_ flux, we performed a molecular ecological network analysis with carbon fixation genes and environmental variables. The resulting network showed typical topological features of scale-free (power law = 0.92), small world (average path distance = 3.49), modular (modularity = 0.64) and hierarchical (average clustering coefficient = 0.21) (Supplementary Table S7). Notably, soil CO_2_ flux showed correlations with *rubisco* genes derived from *Roseovarius, Synechococcus*, and *Nakamurella multipartita*, and *pcc* genes derived from *Mycobacterium* and *Comamonas testosteroni* (**Figure 3A**).

### Nitrogen Cycling Genes

Almost all of nitrogen cycling genes increased in relative abundances with elevations, except that *nosZ* genes (encoding nitrous oxide reductase) were similar across four elevations (Supplementary Figure S6). NO_3_^-^ positively correlated with *amoA* (encoding active site polypeptide of the ammonia monooxygenase)-AOA (ammonia-oxidizing archaea; *r* = 0.51, *P* = 0.087) but negatively correlated with *amoA*-AOB (ammonia-oxidizing bacteria; *r* = -0.60, *P* = 0.044) (**Figures 2F,G**). In addition, NO_3_^-^ positively correlated with a number of nitrogen cycling genes, such as *amoA* genes (*r* = 0.57, *P* = 0.051), *nirK/S* genes (encoding a nitrite reductase, *r* > 0.58, *P* < 0.048), *napA* genes (encoding periplasmic nitrate reductase, *r* = 0.68, *P* = 0.024) and *gdh* genes (encoding glutamate dehydrogenase, *r* = 0.60, *P* = 0.046), and ammonia (NH_4_^+^) negatively correlated with *nirK/S* genes (*r* < -0.58, *P* < 0.046) (Supplementary Table S8). The ratio of *nirK* to *nirS* gene abundances positively correlated with NH_4_^+^ (*r* = 0.59, *P* = 0.046) and negatively correlated with moisture (*r* = -0.46, *P* = 0.123) (Supplementary Table S8). N_2_O flux was positively correlated with *nosZ* gene derived from *Roseobacter denitrificans OCh 114* (*r* = 0.71, *P* = 0.034) and another two probes derived from uncultured bacteria (*r* > 0.69, *P* < 0.036) (Supplementary Table S9), and negatively correlated with *norB* gene derived from *Moraxella catarrhalis* (*r* = -0.88, *P* = 0.002) (Supplementary Table S9).

The association network with *amoA* genes and environmental variables showed topological features of scale-free, small world, modular and hierarchical (Supplementary Table S7). Notably, there was a positive correlation between N_2_O and *amoA* gene derived from *Pseudomonas* (**Figure 3B**), which was abundant in Tibet (Zhang et al., 2007; Tong et al., 2008). In addition, soil pH was negatively correlated with *amoA* gene derived from *Aurantimonas*.

### Stress Response Genes

Induced under cold environment, cold shock proteins help maintain cell phenotypes and viabilities through preventing damage of ice crystal (Willimsky et al., 1992). In our study, increased cold shock genes of *cspA* and *cspB* (encoding cold shock proteins) along the altitudinal gradient suggested stronger capability of microorganisms in acclimation of cold environments (Supplementary Figure S7A). In addition, we found that soil temperature correlated with *cspA* genes (*r* = -0.74, *P* = 0.010) and *cspB* genes (*r* = -0.90, *P* < 0.001) (Supplementary Table S8).

Relative abundances of genes associated with oxygen limitation, radiation stress and mercury generally increased with elevations (Supplementary Figures S7B,C,E), such as *arcA* genes (encoding the cytosolic response regulator) and *arcB* genes (encoding the membrane-bound senor kinase) derived from *Shewanella, Vibrio, Nitrosospira*, and *Oxalobacter*. In contrast, genes associated with glucose limitation were similar in relative abundances (Supplementary Figure S7D). We found that TOC_10-20 cm_ was positively correlated with *mer* genes encoding a mercury-resist operon (*r* = 0.85, *P* = 0.001) (**Figure 2H**). Soil pH was negatively correlated with *mer* (*r* = -0.78, *P* = 0.001) and *merG* (*r* = -0.64, *P* = 0.021) (Supplementary Table S8).

## Discussion

### The Effect of Grazing on Microbial Biogeographic Pattern

It has been shown that soil microbial biomass, taxonomic compositions, and bacterial diversities differed along the altitudinal gradient (Faoro et al., 2010; Fu et al., 2012). Our studies provided evidence that functional gene structures of soil microbial communities also differed along the altitudinal gradient (Supplementary Table S3). We observed increased soil moisture, TN_10-20 cm_ and NO_3_^-^ at higher elevations (**Table 1**), which could provide more electron acceptors and available substrates to stimulate elemental cycling (Xu et al., 2014). In addition, it has been shown that water-extractable organic carbon, microbial biomass carbon, and carbon storage increased with elevations (Pabst et al., 2013; Tsui et al., 2013). Therefore, the increased pattern of microbial diversity could mainly be attributed to the more resources at higher elevations.

The diversity patterns of microbial communities in this study differed from those in enclosed grasslands without livestock grazing using the same data analysis method (Yang et al., 2014). We found microbial functional diversity increased with elevations (Supplementary Table S2). However, microbial functional diversity showed a humpback shape in enclosed grasslands, suggesting that land uses imposed a significant impact on soil microbial communities. Environmental stresses may not contribute to differences in microbial biogeographic patterns, as genes associated with cold shock also increased with elevations in the enclosed grassland. Rather, we speculate that differences in soil nutrient inputs could lead to differences in microbial communities. Animal excreta from livestock provide available nitrogen to vegetation and microbial communities and consequently stimulate nitrogen cycling (Kohler et al., 2005; Ambus et al., 2007). Aboveground vegetation grazed by livestock could reduce litter input to soil, which ameliorates nitrogen limitation for microbial communities because vegetation C/N ratios are higher. These two reasons could collectively stimulate substrate availability to soil microbial communities in pastures.

Livestock grazing could result in more homogenous soil environments because of soil compaction induced by trampling of livestock (Zhao et al., 2007). Similarly, we found that variations of environmental variables in grazing sites, such as NH_4_^+^ (1.34–2.89 mg kg^-1^), soil inorganic nitrogen (SIN; 2.88–3.62 mg kg^-1^) and vegetation species numbers (19.46–26.26), were smaller than variations of NH_4_^+^ (1.5–3.7 mg kg^-1^), SIN (2.88–4.63 mg kg^-1^) and vegetation species numbers (13.67–25.00) in enclosed grasslands (**Table 1**). At the functional gene level, the range of Shannon diversities (10.27–10.58) was less than half of previous observations at the enclosed grassland (9.95–10.65) (Yang et al., 2014), suggesting the important role of livestock grazing on microbial diversities. However, we could not exclude the possibility that some functional genes in soils might not be represented on GeoChip, which would lead to underestimation of microbial functional diversities (Zhou et al., 2015a). Notably, GeoChip is also limited in discovering novel genes in the environment and subjected to errors derived from cross hybridization.

Field simulation studies showed that soils exposed to warmer climates decreased functional diversities (Yue et al., 2015). In addition, warming decreased microbial diversities under normal precipitation conditions (Sheik et al., 2011). It is likely that dominant species would be more advantageous while rare species would extinct, resulting in decreased diversities (Fussmann et al., 2014). Alternatively but not exclusively, warming might increase nutrient availability (Rinnan et al., 2008). Since our results show that microbial functional diversities are higher in colder environments, we predict that warming in Tibetan pastures will decrease microbial functional diversities and shift soil carbon and nitrogen cycling. However, given strong metabolic flexibility and adaptability of soil microbial communities (Bardgett and van der Putten, 2014), it remains to be an open question whether warming will exert permanent effects on functional potentials of microbial communities and other parts of the biosphere (Lau and Lennon, 2011).

### Linkages between Vegetation and Microbial Communities

Unique vegetation species number increased with elevations between 1000 and 4000 m asl in the Tibetan Plateau (Vetaas and Grytnes, 2002). Similarly, we found that numbers of unique genes, which are genes detected only at one elevation, increased with elevations (Supplementary Table S5). The co-occurrence diversification of vegetation and microbes at higher elevations can be attributed to stronger environmental selective forces (Hanson et al., 2012), which was supported by revealing seven environmental variables explaining a total of 82.64% variations of functional gene structure (Supplementary Figure S4) and strong correlation between environmental variables and functional genes (**Table 2**). In contrast, evolutionary or ecological drift might play a minor role.

The tight linkages between vegetation and microbial communities, as indicated by CCA (Supplementary Figure S3) and Mantel tests (**Table 2**), can result from complex interactions between them. Vegetation root exudates and litter input could offer diverse organic resources to influence soil microbial communities (Wardle et al., 2004; Chu et al., 2014), while microbial communities provide feedbacks to vegetation through altering nutrient availability (Lau and Lennon, 2011). In this study, aboveground vegetation biomass correlated with microbial community structures (*r* = 0.52, *P* < 0.010) (**Table 2**), which has been observed elsewhere (Yang et al., 2013; Yue et al., 2015). In contrast, vegetation diversity was not correlated with microbial community structures (*r* = 0.14, *P* > 0.050) (**Table 2**), which could be explained by recent observations that vegetation diversity could predict beta but not alpha diversity of microbial communities in grasslands (Prober et al., 2015). More aboveground vegetation biomass confers more litter and root exudate to soil, which consequently changes C/N ratio of substrates for microbial utilization and alters microbial communities.

### C Cycling Genes

Negative correlations between CO_2_ flux and carbon fixation genes (**Figures 2C–E**) suggested that changes in CO_2_ flux might be attributed to anaerobic acetyl-CoA pathway, reverse TCA cycle and hydroxypropionate pathways. In contrast, Calvin-Benson-Bassham cycle, as indicated by microbial *rubisco* genes encoding an enzyme coupling oxygenolysis of ribulose-1, 5-bisphophate and CO_2_ reduction, were similar along the altitudinal gradient. However, this observation does not refute the possibility that *rubisco* genes play a substantial role in soil CO_2_ flux. Recently, microbial *rubisco* gene abundance and enzyme activities were shown to be high in Tibetan grasslands (Guo et al., 2015). In addition, we have identified correlations between CO_2_ flux and *rubisco* genes derived from *Roseovarius, Synechococcus*, and *N. multipartita* by the association network analysis. Therefore, individual functional species possessing *rubisco* genes might contribute to variations of CO_2_ flux, despite the lack of overall changes in total abundance of *rubisco* genes.

Higher soil moisture strengthens substrate utilization of microbial communities (Chen et al., 2007). Furthermore, TOC, readily mineralizable carbon, water-soluble organic carbon and reducing sugar carbon increase when soil water-holding capability varies in 20–60% (Hassan et al., 2015), owing to increase in microbial accessibility to soluble nutrients, especially autotrophic carbon, ammonia and nitrate (Bell et al., 2008; Van Horn et al., 2014). Accordingly, we observed an increase of carbon degradation genes (Supplementary Figure S5A).

Soil moisture also has a strong effect on net soil CH_4_ uptake rate, which is the balance between CH_4_ production and oxidation (Shrestha et al., 2012). As CH_4_ production and oxidation are two tightly intertwined processes (Inagaki et al., 2004), it is no surprise to note an increase in relative abundances of both CH_4_ production and oxidation genes with elevations and soil moisture (Supplementary Figure S5C). Similarly, more CH_4_ and available substrates induced by soil moisture can stimulate microbial methanotrophs as well as CH_4_ oxidation gene abundance (Shrestha et al., 2012). Conversely, CH_4_ oxidation stimulates the growth of methanogenic bacteria by supplying energy (Hoehler et al., 1994).

### N Cycling Genes

Many studies used *nirS* and (or) *nirK* genes as functional markers to profile diversities or structures of denitrifiers because nitrite reductase is the rate-limiting step of denitrification (Braker et al., 1998; Liu et al., 2003). The ratio of *nirK* to *nirS* gene abundance negatively and positively correlated with soil moisture (*r* = -0.46, *P* = 0.123) and NH_4_^+^ (*r* = 0.59, *P* = 0.046), respectively (Supplementary Table S8), which was consistent with a recent finding that water logging decreased the *nirK*/*nirS* ratio (Yoshida et al., 2009). In addition, both NO_3_^-^ and NH_4_^+^ are associated with community compositions of *nirS*-type denitrifiers (Zheng et al., 2015).

The correlation between *amoA* and NO_3_^-^ (*r* = 0.57, *P* = 0.051) has been similarly documented (Ding et al., 2015). Intriguingly, *amoA*-AOB negatively correlated with NO_3_^-^ (*r* = -0.60, *P* = 0.044) and *amoA*-AOA positively correlated with NO_3_^-^ (*r* = 0.51, *P* = 0.087) (**Figures 2F,G**), which could be attributed to different roles of ammonia monooxygenase, physiological pathways and electron transport mechanisms in AOA and AOB (Hatzenpichler, 2012). Consistently, it was shown in groundwater that nitrate positively correlated with copy numbers of *amoA*-AOA and negatively correlated with those of *amoA*-AOB (Qin et al., 2014). The positive correlations between NO_3_^-^ and *hzo* and *napA* genes provide further evidence to the finding that NO_3_^-^ can regulate the anammox and dissimilarity nitrate reduction processes (Silver et al., 2001; Trimmer et al., 2005).

### Heavy Metal

Livestock manure was one of heavy metal sources to soil, including mercury (Nicholson et al., 2003; Wang et al., 2016). At higher elevations, decreased turnover rate of soil organic carbon could accelerate mercury accumulation and cold temperature could decrease mercury evasion, causing the increasing abundance of *mer* genes (Zhou et al., 2015b). Consistently, *mer* gene positively correlated with TOC_10-20 cm_ (*r* = 0.85, *P* = 0.001) (**Figure 2H**), which might be attributed to mercury affinity to organic matter (Zhou et al., 2015b). In addition, the negative correlations between pH and *mer* (*r* = -0.78, *P* = 0.001) and *merG* (*r* = -0.64, *P* = 0.021) genes indicated that soil acidification constrained Hg volatilization (Supplementary Table S8).

## Conclusion

This study represents an in-depth investigation of soil microbial functional gene profiles in an alpine pasture, based on GeoChip. Compared to enclosed grassland, stronger elevation-decay relationships of microbial functional genes were detected, which could be ascribed to strong environment selection in the alpine environment. By focusing on microbial functional genes, our study provides valuable insights for understanding microbe-mediated mechanisms of soil biogeochemical cycling.

## Data Accessibility

GeoChip 4.0 data is available online^1^ with the accession number GSE52425.

## Author Contributions

This study was conceived and led by SW, JZ, and YY. YY, SW, QL, and XL contributed to GeoChip experiments and environmental measurements. QQ, MZ, and YG analyzed data. QQ and MZ led the efforts to synthesize the data and write manuscript. YY, YW, BG, GL, and XM revised this manuscript. All authors read and approved the final manuscript.

## Conflict of Interest Statement

The authors declare that the research was conducted in the absence of any commercial or financial relationships that could be construed as a potential conflict of interest. The reviewer WK declared a shared affiliation, though no other collaboration, with the author SW and additionally different institutes to the authors QL, XL to the handling Editor, who ensured that the process nevertheless met the standards of a fair and objective review. The reviewer SW declared a shared affiliation, though no other collaboration and different institutes, with several of the authors SW, QL, XL to the handling Editor, who ensured that the process nevertheless met the standards of a fair and objective review.

## Footnotes

1 http://www.ncbi.nlm.nih.gov/geo/
