# Supplementary material for: The Biogeographic Pattern of Microbial Functional Genes along an Altitudinal Gradient of the Tibetan Pasture
Source: Front Microbiol. 2017 Jun 13;8:976. doi: 10.3389/fmicb.2017.00976 (PMC5468456; doi:10.3389/fmicb.2017.00976)
Supplement: Supplementary file 1 [file Data_Sheet_1.docx]

**Supplemental materials**

**The biogeographic pattern of microbial functional genes along an altitudinal gradient of the Tibetan pasture**

Qi Qi^1,†^, Mengxin Zhao^1,†^, Shiping Wang^2, 3^, Xingyu Ma^1^, Yuxuan Wang^4,5^, Ying Gao^1^, Qiaoyan Lin^6^, Xiangzhen Li^7^, Baohua Gu^8^, Guoxue Li^9^, Jizhong Zhou^1,10,11,12^ and Yunfeng Yang^1,*^

^1^State Key Joint Laboratory of Environment Simulation and Pollution Control, School of Environment, Tsinghua University, Beijing, China, ^2^Key Laboratory of Alpine Ecology and Biodiversity, Institute of Tibetan Plateau Research, Chinese Academy of Sciences, Beijing, China, ^3^CAS Center for Excellence in Tibetan Plateau Earth Science, Beijing, China, ^4^Department of Earth System Sciences, Tsinghua University, Beijing, China, ^5^Department of Earth and Atmospheric Sciences, University of Houston, Houston, TX, USA, ^6^Key Laboratory of Adaption and Evolution of Plateau Biota, Northwest Institute of Plateau Biology, Chinese Academy of Sciences, Xining, Qinghai, China, ^7^Key Laboratory of Environmental and Applied Microbiology; Environmental Microbiology Key Laboratory of Sichuan Province, Chengdu Institute of Biology, Chinese Academy of Sciences, Chengdu, Sichuan, China, ^8^Environmental Sciences Division, Oak Ridge National Laboratory, Oak Ridge, Tennessee, USA, ^9^College of Resources and Environmental Science, China Agricultural University, Beijing, China, ^10^Institute for Environmental Genomics and Department of Microbiology and Plant Biology, University of Oklahoma, Norman, Oklahoma, USA, ^11^Earth Sciences Division, Lawrence Berkeley National Laboratory, Berkeley, California, USA and ^12^Collaborative Innovation Center for Regional Environmental Quality, School of Environment, Tsinghua University, Beijing, China

^†^These authors contributed equally to this work.

^*^Address correspondence to Yunfeng Yang, E-mail: yangyf@tsinghua.edu.cn; Phone: +86-010-62784692; Fax: +86-010-62785687;

**Fig. S1** Percentages of genes detected at one (unique), two, three and four (ubiquitous) elevations.


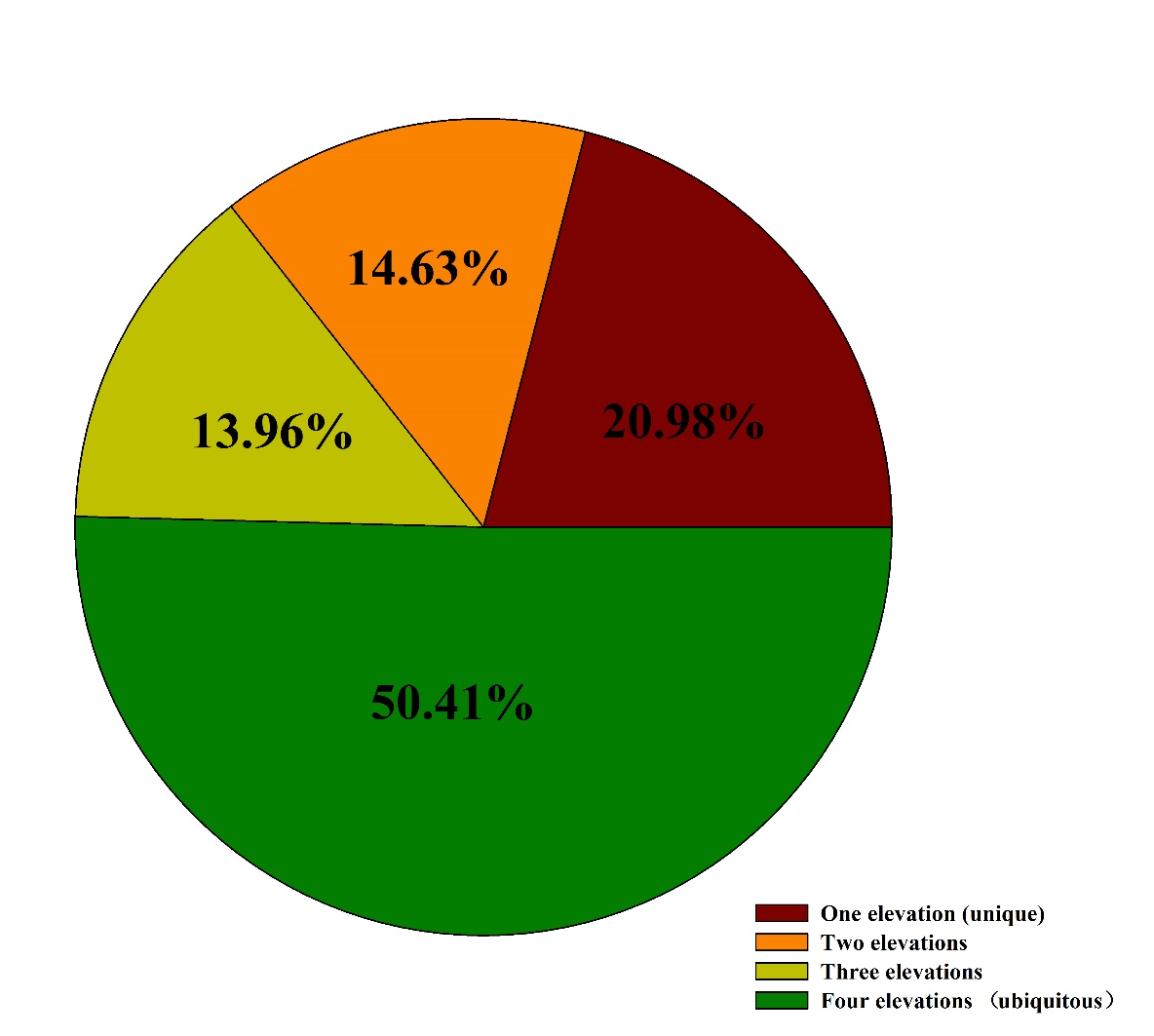


**Fig. S2** Correlations between geographical distances and numbers of shared genes among different soil samples. Pearson correlation and TDIST tests were used to calculate *r* and *P* values, respectively.


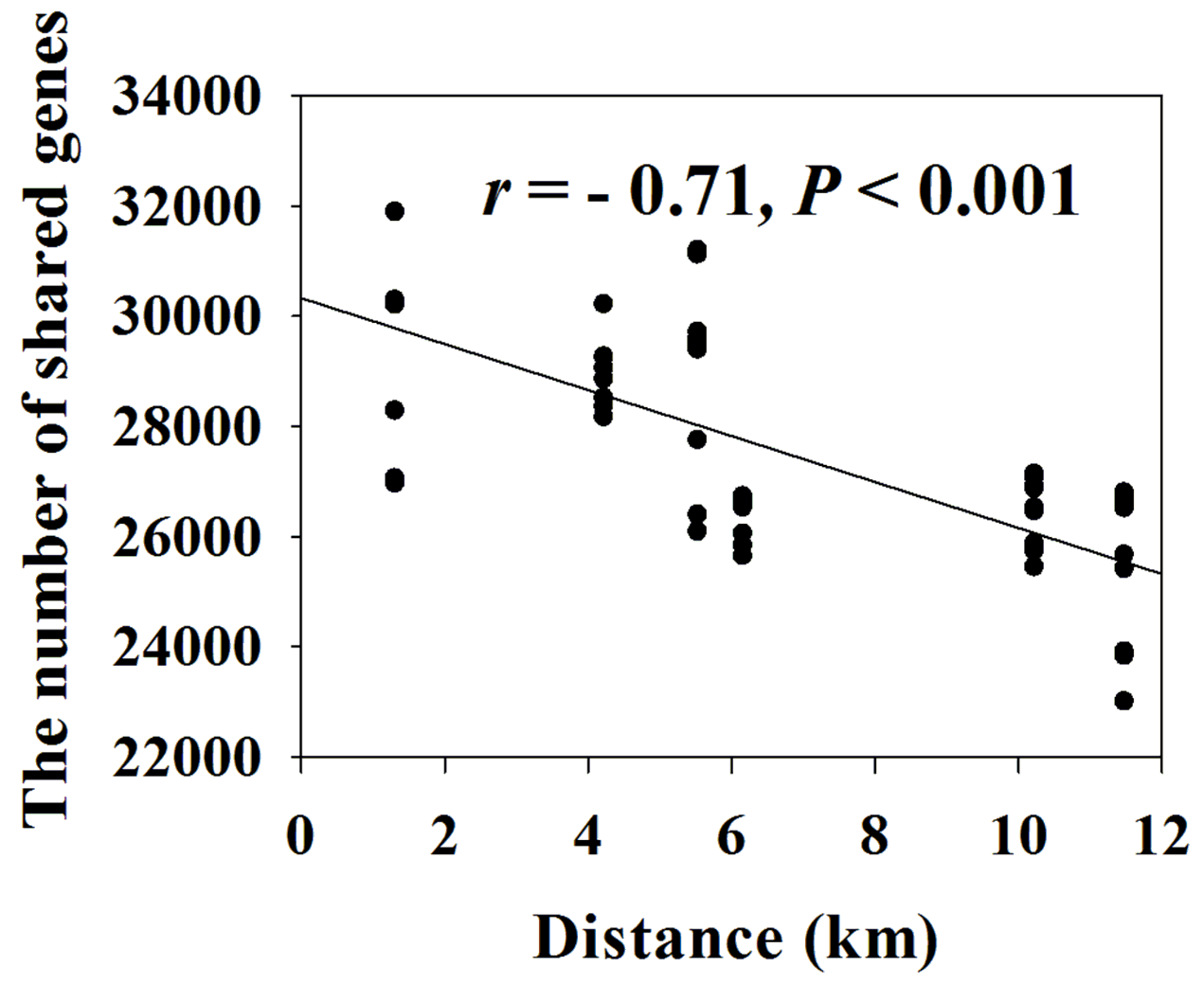


**Fig. S3** The canonical correspondence analysis (CCA) between GeoChip data and environmental variables. Symbols represent GeoChip data and arrows represent seven environmental variables (soil temperature (T), pH, nitrate (NO_3_^-^), total phosphorus measured at 10-20cm depth (TP_10-20 cm_), aboveground vegetation biomass, species number and diversity).

**

**

**Fig. S4** Variation partitioning analysis that partitions environmental variables into three groups: (A) soil temperature (T); (B) soil pH, nitrate (NO_3_^-^) and total phosphorus measured at 10-20 cm depth (TP_10-20 cm_); (C) aboveground vegetation biomass, species number and diversity. Non-overlap area of each circle and overlap area of adjacent circles mean individual effects of each group and joint effects of adjacent groups, respectively. The unexplained microbial community structure showed at the bottom.


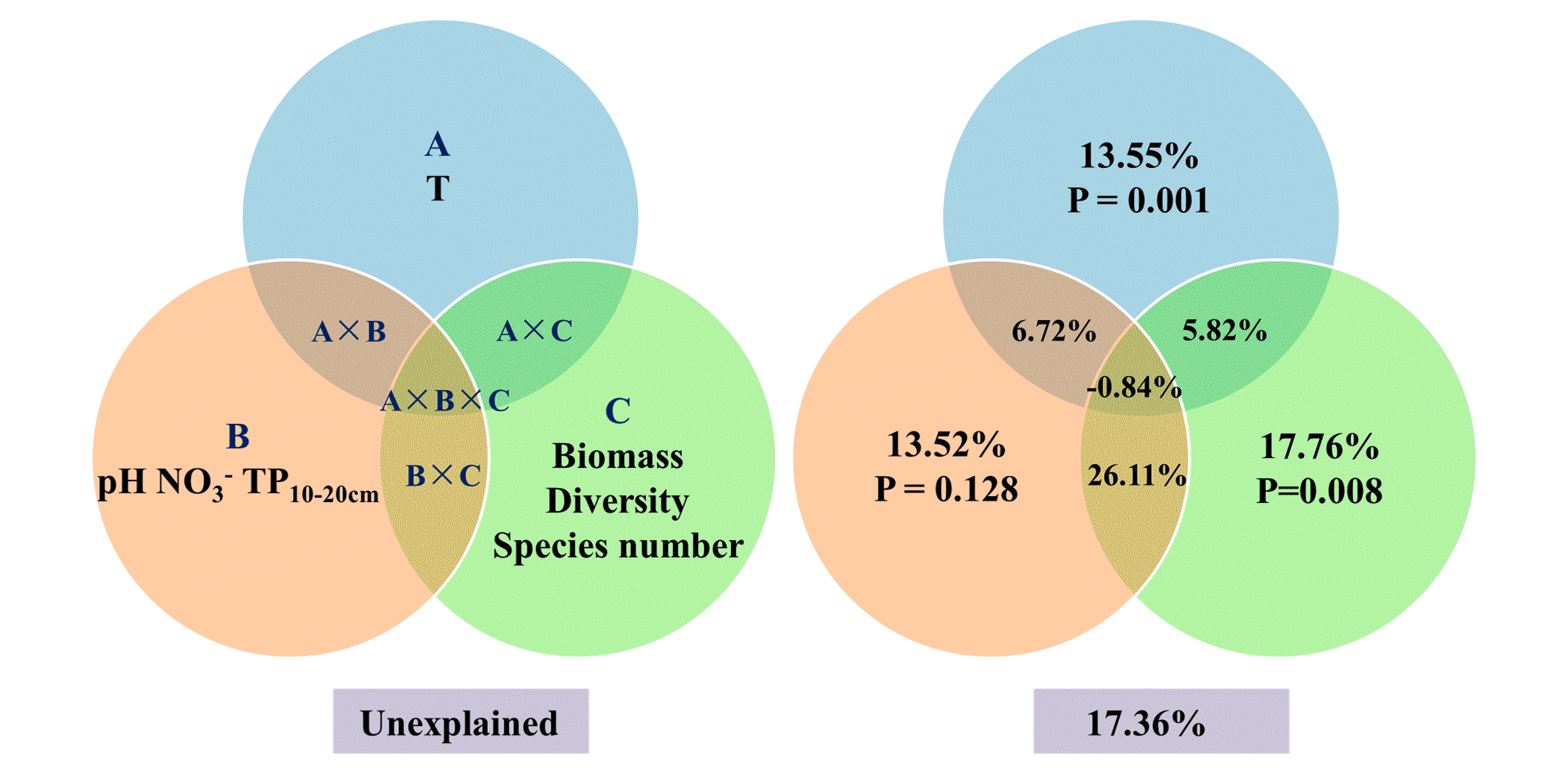


**Fig. S5** Relative abundances of genes associated with (A) selected carbon degradation, (B) carbon fixation and (C) methane. All data are presented as mean ±s.e. calculated by biological triplicates. Significant (*P* < 0.050) differences among elevations are indicated by different alphabetic letters above the bars.








**Fig. S6** Relative abundances of genes associated with nitrogen cycling. All data are presented as mean ±s.e.. Alphabetic letters above the bars represent significant (*P* < 0.050) differences among elevations.

**

**

**Fig. S7** Relative abundances of genes associated with (A) cold shock, (B) oxygen limitation, (C) radiation, (D) glucose limitation and (E) mercury resistance. All data are presented as mean ±s.e. calculated by biological triplicates. Significant (*P* < 0.050) differences among elevations are indicated by different alphabetic letters above the bars.








**SUPPORTING TABLES**

**Table S1**. Correlations among total organic carbon, total nitrogen and total phosphorus.

|  | *r* |
| --- | --- |
| TOC_0-10 cm_^a^ - TN_0-10 cm_ | 0.88**^b^ |
| TOC_0-10 cm_ - TP_0-10 cm_ | 0.81** |
| TOC_0-10 cm_ - TN_10-20 cm_ | 0.87** |
| TOC_10-20 cm_ - TP_0-10 cm_ | 0.63* |
| TN_0-10 cm_ - TP_0-10 cm_ | 0.96*** |

^a^Abbreviations: TOC - total organic carbon; TN - total nitrogen; TP - total phosphorus; 0-10 cm and 10-20 cm - soil variables measured at 0-10 and 10-20 cm depth, respectively.

^b^**P* < 0.050, ***P* < 0.010, ****P* < 0.001

**Table S2.** The richness, diversity and evenness indices of functional genes at each elevation.

|  | 3200m | 3400m | 3600m | 3800m |
| --- | --- | --- | --- | --- |
| Richness^a^ | 31528 | 37752 | 42226 | 43816 |
| Shannon index | 10.27c^b^ | 10.44b | 10.54a | 10.58a |
| Simpson index | 28900.04c | 34076.60b | 37935.60a | 39470.65a |
| Pielou evenness | 0.99961a | 0.99962a | 0.99967a | 0.99959a |
| Simpson evenness | 0.99210a | 0.99209a | 0.99297a | 0.99140a |

^a^Detected gene number.

^b^Significant (*P*<0.05) differences among elevations are indicated by different alphabetic letters.

**Table S3**. Differences of microbial communities among elevations as examined by the dissimilarity tests of *adonis*.

| Sites (m) | Statistic *R*^2^ | *P* |
| --- | --- | --- |
| 3200-3400 | 0.62 | 0.031 |
| 3200-3600 | 0.73 | 0.001 |
| 3200-3800 | 0.81 | 0.001 |
| 3400-3600 | 0.66 | 0.001 |
| 3400-3800 | 0.70 | 0.001 |
| 3600-3800 | 0.73 | 0.001 |

**Table S4** The elevation-decay relationships of different functional categories.

| Group | *r* of correlation | *P* of correlation^a^ | *z* | *t* | *n* | *P^b^* | Lower 95% CI | | Upper 95% CI^c^ |
| --- | --- | --- | --- | --- | --- | --- | --- | --- | --- |
| Whole microbial community | -0.80 | <0.001 | 0.0093 | -209.6 | 999 | <0.001 | -1.22E-04 | 5.30E-05 | |
| Carbon cycling | -0.85 | <0.001 | 0.0076 | -216.7 | 999 | <0.001 | -5.04E-05 | 8.76E-05 | |
| Carbon fixation | -0.83 | <0.001 | 0.0061 | -213.3 | 999 | <0.001 | -1.04E-04 | 8.87E-06 | |
| Carbon degradation | -0.86 | <0.001 | 0.0078 | -218.7 | 999 | <0.001 | -2.20E-05 | 1.16E-04 | |
| Methane | -0.73 | <0.001 | 0.0111 | -179.0 | 999 | <0.001 | -6.42E-05 | 1.79E-04 | |
| Nitrogen cycling | -0.83 | <0.001 | 0.0065 | -206.6 | 999 | <0.001 | -7.92E-05 | 4.37E-05 | |
| Ammonification | -0.82 | <0.001 | 0.0062 | -204.7 | 999 | <0.001 | -4.98E-05 | 6.82E-05 | |
| Anammox | -0.75 | <0.001 | 0.0127 | -184.4 | 999 | <0.001 | -1.11E-04 | 1.58E-04 | |
| Assimilatory N reduction | -0.83 | <0.001 | 0.0065 | -208.8 | 999 | <0.001 | -4.26E-05 | 8.02E-05 | |
| Denitrification | -0.78 | <0.001 | 0.0058 | -194.0 | 999 | <0.001 | -6.14E-06 | 1.10E-04 | |
| Dissimilatory N reduction | -0.83 | <0.001 | 0.0090 | -210.0 | 999 | <0.001 | -8.62E-05 | 8.29E-05 | |
| Nitrogen fixation | -0.80 | <0.001 | 0.0055 | -191.5 | 999 | <0.001 | -1.81E-05 | 9.33E-05 | |
| Nitrification | -0.81 | <0.001 | 0.0085 | -202.3 | 999 | <0.001 | -5.09E-05 | 1.13E-04 | |

^a^*P* value of Pearson correlation.

^b^*P* value of one sample t-test.

^c^CI, confidence interval.

**Table S5.** The percentages of unique genes to detected genes and numbers of unique genes at each elevation.

| Category | 3200m | 3400m | 3600m | 3800m |
| --- | --- | --- | --- | --- |
| Antibiotic resistance | 0.008 (10)^a^ | 0.025 (36) | 0.131 (216) | 0.123(197) |
| Bacteria phage | 0.016 (4) | 0.030 (4) | 0.167 (69) | 0.190 (80) |
| Bioleaching | 0.005 (1) | 0.038 (11) | 0.158 (57) | 0.202 (77) |
| Carbon cycling | 0.006 (25) | 0.026(131) | 0.100 (552) | 0.129 (742) |
| Energy process | 0.003 (1) | 0.017 (7) | 0.101 (47) | 0.131 (66) |
| Mental resistance | 0.006 (23) | 0.023 (100) | 0.089 (430) | 0.125 (639) |
| Nitrogen cying | 0.006 (19) | 0.025 (89) | 0.096 (370) | 0.114 (453) |
| Complex carbon degradation | 0.004 (33) | 0.021 (193) | 0.062 (586) | 0.112 (1137) |
| Phosphorus  cyling | 0.004 (2) | 0.017 (10) | 0.119 (81) | 0.164 (118) |
| Stress | 0.007 (53) | 0.027 (243) | 0.123 (1276) | 0.143 (1514) |
| Sulphur cyling | 0.009 (11) | 0.031 (44) | 0.107 (168) | 0.149 (244) |
| Virulence | 0.005 (6) | 0.026 (38) | 0.131 (234) | 0.139 (249) |

^a^Values in parentheses are endemic gene numbers.

**Table S6**. The number of shared genes and the percentage of shared to detected genes between two elevations.

| Sites | 3200m | 3400m | 3600m | 3800m |
| --- | --- | --- | --- | --- |
| 3200m |  | 29669 (56.12)^a^ | 29832 (56.41) | 28852 (54.57) |
| 3400m |  |  | 33250 (62.89) | 33590 (63.54) |
| 3600m |  |  |  | 34614 (65.47) |
| 3800m |  |  |  |  |

^a^Values in parentheses are percentages.

**Table S7.** Major topological properties of the molecular ecological networks of carbon fixation genes and *amoA* genes.

| Community | Carbon fixation genes | *amoA* genes |
| --- | --- | --- |
| Number of genes used for network construction | 684 | 371 |
| Network size (n) | 350 | 250 |
| Modularity (number of modules) | 0.637 (45) | 0.626 (25) |
| *R*^2^ of power-law | 0.923 | 0.923 |
| Average connectivity (avgK) | 3.943 | 4.696 |
| Average clustering coefficient (avgCC) | 0.207 | 0.267 |
| Average path distance (GD) | 3.488 | 3.479 |
| Harmonic geodesic distance (HD) | 6.361 | 5.328 |
| Centralization of degree (CD) | 0.046 | 0.082 |
| Centralization of betweenness (CB) | 0.100 | 0.119 |
| Centralization of stress centrality (CS) | 0.513 | 0.537 |
| Maximal eigenvector centrality | 0.265 | 0.272 |
| Centralization of eigenvector centrality (CE) | 0.244 | 0.244 |
| Density (D) | 0.011 | 0.019 |
| Connectedness (Con) | 0.668 | 0.726 |
| Efficiency | 0.987 | 0.979 |

**Table S8.** The correlations between relative abundances of functional genes and environmental variables.

| Number | Environmental variable | Gene name | *r* | *P* |
| --- | --- | --- | --- | --- |
| 1 | NO_3_^—^ | *nirK* | 0.62 | 0.041 |
| 2 | NO_3_^—^ | *nirS* | 0.58 | 0.048 |
| 3 | NO_3_^—^ | *norB* | 0.74 | 0.014 |
| 4 | NO_3_^—^ | *napA* | 0.68 | 0.024 |
| 5 | NO_3_^—^ | *amoA* | 0.57 | 0.051 |
| 6 | NO_3_^—^ | *gdh* | 0.60 | 0.046 |
| 7 | NO_3_^—^ | *hzo* | 0.61 | 0.041 |
| 8 | NO_3_^—^ | *NirB* | 0.69 | 0.022 |
| 9 | NH_4_^+^ | *nirK* | -0.58 | 0.046 |
| 10 | NH_4_^+^ | *nirS* | -0.64 | 0.035 |
| 11 | NH_4_^+^ | *NiR* | -0.59 | 0.045 |
| 12 | NH_4_^+^ | *nirK/nirS* | 0.59 | 0.046 |
| 13 | Moisture | *nirK/nirS* | -0.46 | 0.123 |
| 14 | T | *cspA* | -0.74 | 0.010 |
| 15 | T | *cspB* | -0.90 | <0.001 |
| 16 | pH | *mer* | -0.78 | 0.001 |
| 17 | pH | *merG* | -0.64 | 0.021 |

^a^Abbreviation: T - soil temperature

**Table S9.** The correlations between relative abundances of functional probes and N_2_O flux.

| Genebank number | Environemntal variables | Gene name | Organism | *r* | *P* |
| --- | --- | --- | --- | --- | --- |
| 83972416 | N_2_O flux | *nosZ* | uncultured alpha proteobacterium | 0.81 | 0.008 |
| 55469821 | N_2_O flux | *nosZ* | uncultured bacterium | 0.69 | 0.036 |
| 110678855 | N_2_O flux | *nosZ* | *Roseobacter denitrificans OCh 114* | 0.71 | 0.034 |
| 157285377 | N_2_O flux | *nosZ* | uncultured bacterium | -0.87 | 0.002 |
| 215512185 | N_2_O flux | *norB* | *Moraxella catarrhalis* | -0.88 | 0.002 |
